# Social Determinants, Data Science, and Decision Making: The 3-D Approach to Achieving Health Equity in Asia

**DOI:** 10.34133/2022/9805154

**Published:** 2022-02-21

**Authors:** Luxia Zhang, Sabina Faiz Rashid, Gabriel Leung

**Affiliations:** ^1^National Institute of Health Data Science, Peking University, Beijing, China; ^2^Advanced Institute of Information Technology, Peking University, Hangzhou, Zhejiang Province, China; ^3^Renal Division, Department of Medicine, Peking University First Hospital, Beijing, China; ^4^BRAC James P Grant School of Public Health, BRAC University, Dhaka, Bangladesh; ^5^WHO Collaborating Centre for Infectious Disease Epidemiology and Control, School of Public Health, LKS Faculty of Medicine, The University of Hong Kong, Hong Kong Special Administrative Region, China; ^6^Laboratory of Data Discovery for Health, Hong Kong Science and Technology Park, Hong Kong Special Administrative RegionChina

Improving population health by creating more equitable health systems is a major focus of health policy and planning today. However, before we can achieve equity in health, we must first begin by leveraging all we have learned, and are continuing to discover, about the many social, structural, and environmental determinants of health. We must fully consider the conditions in which people are born, grow, learn, work, play, and age. The study of social determinants of health has made tremendous strides in recent decades. At the same time, we have seen huge advances in how health data are collected, analyzed, and used to inform action in the health sector. It is time to merge these two fields, to harness the best from both and to improve decision-making to accelerate evidence-based action toward greater health equity.

This is the crux of a recently published report (https://3dcommission.health/report) by the Commission on Health Determinants, Data and Decision-Making (3-D Commission), an initiative by the Rockefeller Foundation and Boston University School of Public Health. Over the past two years, the 3-D Commission has delved into the key social and economic drivers that influence health outcomes and explored how data science and the social determinants of health should be integrated into decision-making processes. The key concepts of the 3-D Commission are especially important for Asian countries. Rapid demographic, social, and economic changes in Asia are prompting multiple health challenges including the spread of infectious diseases and the rise of chronic noncommunicable diseases. Furthermore, access to healthcare is still an issue for the marginalized communities within Asian countries. Those challenges, together with the rising affluence and expectations for better health services, constitute the need for transformation of healthcare system. Government officials should aim to reach an ideal where the data on the social determinants of health is utilized in decision-making and policies are implemented which contribute to the achievement of health equity. 

The findings of the 3-D Commission’s final report (https://3dcommission.health/report) are centered around the need to consider the full spectrum of factors, barriers, and opportunities for using data on determinants and to use those data more systematically to inform policies and practices aimed at improving health across societies at different points in the development spectrum. The impacts of social determinants are long term, dependent on context, and intersect with other variables. Given the lengthy causal pathway between exposure to certain social factors and health outcomes—and all the intervening factors along the way—it can be difficult to quantify their effects. This is where the use of data becomes essential. Policymakers must continually connect the dots between epidemiological data and social determinant data to understand the health challenges they are facing more fully. 

The 3-D Commission report (https://3dcommission.health/report) offers a roadmap for scholars, practitioners, researchers, and policymakers alike, with recommendations in three focus areas—political will, technical capacity, and community engagement—to drive results. Its recommendations are rooted in the following six principles for action to help shape the policy response and practical action in the face of health crises:

(i) Evidence-informed decision-making to promote healthy societies needs to go beyond healthcare and incorporate data on the broader determinants of health
(ii) All decisions about investments in any sector need to be made with health as a consideration
(iii) Decision-making that affects population health needs to embrace health equity—while also acknowledging potential tradeoffs between short- and long-term costs and benefits
(iv) All available data resources on the determinants of health should be used to inform decision-making about health
(v) Data on the social determinants of health should contribute to better, more transparent, and more accountable governance
(vi) Evidence-informed decision-making to promote healthy societies needs to be participatory and inclusive of multiple and diverse perspectives

In Asian countries, there are certain distinctive features regarding the 3-D (Health Determinants, Data and Decision-Making) elements, including the cultural values’ impact on decision-making process, the supply constraints of healthcare resources, the substantial variation in data ecosystem of health and beyond, and the consumer-oriented nature of healthcare system. Hence, the roadmap of the 3-D Commission needs to be localized in Asia. The following example from Bangladesh during COVID-19 pandemic reflects the importance of harnessing multisource data for decision-making and including health considerations for the government.

A team of researchers from the James P. Grant School of Public Health (http://bracjpgsph.org), at Brac University in Bangladesh, has been studying the impacts of COVID-19-induced lockdowns on people who live and work in the slums of Dhaka to capture their lived experiences and to understand their needs better. The team conducted a rapid large-scale urban/rural survey and longitudinal research in urban slums to assess how the pandemic has affected consumption, income, health coping strategies, psychological well-being, and other issues. The findings from these studies represent the type of data we need more of—and need to pay closer attention to—to ensure that public health interventions reflect the socio-economic realities of life for thosewho face the greatest health risks. It is what decision makers need to assess those short- vs. long-term costs and benefits—and to effectively reduce health inequities. 

The government of Bangladesh has introduced an economic stimulus plan to help businesses that have struggled during the pandemic [1]. The government is also working on a new support program for the poor that includes supporting farmers, whom everybody relies on to maintain domestic food supplies. This is a great start, but cross-sector coordination has to work both ways. We must make sure health considerations related to these issues are kept top of mind; that these and other programs are data driven; that the process for developing them is participatory and inclusive; and that they are implemented efficiently and equitably. This is how we build resilience against future crises. And by broadening our vision, we can make meaningful progress for those who need it the most. 
